# Preclinical *ex vivo* IL2RG gene therapy using autologous hematopoietic stem cells as an effective and safe treatment for X-linked severe combined immunodeficiency disease

**DOI:** 10.1016/j.gendis.2024.101445

**Published:** 2024-11-06

**Authors:** Mingfeng Hu, Qiling Xu, Fang Zhang, Karen F. Buckland, Yelei Gao, Weixia Du, Yuan Ding, Lina Zhou, Xiulian Sun, Lijia Ma, Zhiyong Zhang, Xuemei Tang, Xiaodong Zhao, Adrian J. Thrasher, Yunfei An

**Affiliations:** a Department of Pediatric Research Institute, Ministry of Education Key Laboratory of Child Development and Disorders, National Clinical Research Center for Child Health and Disorders (Chongqing), China International Science and Technology Cooperation Base of Child Development and Critical Disorders, Children's Hospital of Chongqing Medical University, Chongqing 400014, China; b Chongqing Key Laboratory of Child Infection and Immunity, Children's Hospital of Chongqing Medical University, Chongqing 400014, China; c Department of Rheumatology and Immunology, Children's Hospital of Chongqing Medical University, Chongqing 400014, China; d UCL Great Ormond Street Institute of Child Health, London WC1N 1EH, UK; e Ubrigene (Beijing) Biosciences Co. Ltd, Beijing 100080, China; f Genome Editing, Westlake Laboratory of Life Sciences and Biomedicine, Hangzhou, Zhejiang 310024, China

**Keywords:** Atypicaldiverse phenotype, Gene therapy, *IL2RG*, Self-inactivating lentiviral vector, X-linked severe combined immunodeficiency disease

## Abstract

X-linked severe combined immunodeficiency disease (X-SCID) is a rare inherited disease caused by mutations in the interleukin 2 receptor subunit gamma gene (*IL2RG*), which encodes the common γ chain protein, a subunit of the receptor for lymphocytes. X-SCID is characterized by profound defects in T-cell, B-cell, and natural killer cell function. Here, we report a Chinese cohort of nine X-SCID patients with six novel *IL2RG* mutations. Among those, the two adolescent patients with an atypical immunotype were confirmed by further analyzing IL-2-JAK-STAT5 signaling, T cell proliferation, and T cell receptor excision circles (Trecs). Interestingly, Bacillus Calmette-Guérin (BCG) disease occurred commonly in this cohort. Although allogeneic hematopoietic stem-cell transplantation is curative for the disease, it is not available to all patients due to the lack of suitable matched donors. Autologous gene therapy using a self-inactivating lentiviral vector (SIN-LV) technology has provided an alternative therapy for such mono-genetic diseases. Here, we performed the pre-clinical studies to assess our SIN-LV carrying *IL2RG* on human ED7R cells deficient in *IL2RG* and CD34^+^ stem cells derived from the bone marrow of a healthy donor and a patient with X-SCID. This work is done complied with the established “Good Manufacturing Practice” (GMP) used in the clinical trials. In addition, a safety study is performed using the transduced CD34^+^ cells implanted into the axilla of nude mice *in vivo*. Overall, our studies have demonstrated the efficiency and safety of SIN-IL2RG-LV, which paves the way for conducting X-SCID gene therapy clinical trials in China in the near future.

## Introduction

X-linked severe combined immunodeficiency disease (X-SCID), which leads to profound defects in the function of T-cell, B-cell, and natural killer (NK) cell functions, is caused by mutations in the interleukin 2 receptor γ-chain gene (*IL2RG*).^1^ The γ-chain (CD132) encoded by *IL2RG* is shared by several hematopoietic cytokine receptors, including the interleukin (IL)-2, IL-4, IL-7, IL-9, IL-15, and IL-21 receptors.^2^^,^^3^ Children with X-SCID may die in the very early years of life due to recurrent infections, and few survive to adolescence ^4^; however, the rapid technological innovation and wide application of next-generation sequencing screening means that an increasing number of atypical patients who are not considered clinically or immunologically “severe” have been reported.

Curative treatment for X-SCID aims to restore immune function; to date, this has been attempted using either allogeneic hematopoietic stem cell transplantation (HSCT) or gene therapy.^5^^,^^6^ Gene therapy, which utilizes autologous cells, can avoid problems related to donor availability/compatibility and graft-versus-host disease. Moloney murine leukemia virus-based gamma retroviral vectors (γRVs) containing wild-type IL2RG cDNA, transcription of which is regulated by endogenous viral long terminal repeat sequences, have been used in gene therapy clinical trials in patients with X-SCID.^7^, ^8^, ^9^ CD34^+^ cells transduced with this vector were infused into patients without pre-conditioning. Most patients showed good long-term T cell immune reconstitution, with an overall survival rate of 90% (18/20); however, six of the patients in the initial trials developed serious adverse events associated with T cell acute lymphoblastic leukemia, caused by the viral enhancer-mediated mutagenesis, at 2–14 years post-gene therapy.^10^, ^11^, ^12^, ^13^, ^14^, ^15^ To improve safety, next-generation self-inactivating (“SIN”) γRVs were developed and used without conditioning to treat patients at five centers in the United States and Europe. In a recent report, after a median follow-up of 7.9 years (2.7–9.3 years), none of the 14 X-SCID patients treated with SIN-γRVs developed leukemia. The study also reported robust T-cell recovery in those patients receiving cells with sufficient transduction.^16^ Safety is further improved using a self-inactivating lentiviral vector (SIN-LV). A recent publication described eight infants with X-SCID treated with SIN-LVs-based gene therapy. Successful immune reconstitution of gene-corrected B, T, and NK cells, as well as improved IgG levels, after short-term follow-up was demonstrated clearly.^17^ Another publication described a phase I/II clinical trial involving five older X-SCID patients treated with SIN-LV after busulfan conditioning.^18^ Clinical benefits were achieved, and no vector-related complications have been reported.

In China, the incidence of X-SCID is quite high. About 300 patients are born every year. Although the overall survival of patients with X-SCID treated by HSCT is over 80%, most patients lack suitable donors and are often compromised by ongoing severe infections, which can be life-threatening. Thus, exploring optimal gene therapy protocols that are suitable for Chinese patients with X-SCID is desirable.

Here, we investigated the clinical, molecular, and immune characteristics of nine patients with X-SCID, all of whom are candidates for inclusion in a clinical study. We identified six novel mutations in *IL2RG*. Here, we report our preclinical study aimed at addressing the efficacy and safety of SIN-LV vector-based gene therapy for X-SCID and confirm the efficiency of this vector in CD34^+^ cells derived from the bone marrow (BM) of a patient with X-SCID.

## Materials and methods

### Patient and study approval

Clinical data and samples from patients and healthy controls were collected at the Children's Hospital of Chongqing Medical University. All legal guardians and control donors provided informed consent in accordance with the Declaration of Helsinki, and the study was approved by the Medical Ethics Committee of the Children's Hospital of Chongqing Medical University (approval number: 030/2018).

### Flow cytometry analysis

Cells were resuspended in phosphate buffer saline solution and incubated with antigen-specific antigens or isotype controls at 4 °C for 30 min. To detect phospho-STAT5 (pSTAT5), peripheral blood mononuclear cells were surfaced stained with anti-CD3, -CD4, -CD8, and -CD56 antibodies and then stimulated with IL-2 (100 ng/mL; PeproTech, New Jersey, USA) at 37 °C for 0, 15, 30, or 60 min. Cell suspensions were then mixed immediately with equal volumes of pre-warmed BD Phosflow Fix Buffer I (BD Biosciences, San Jose, CA, USA) and incubated at 37 °C for 10 min. After the addition of cold BD Phosflow Perm Buffer III (BD Biosciences), the permeabilized cells were washed twice and then stained with phospho-STAT5 for 30 min. Stained cells were washed and events were captured by a flow cytometer (FACS Canto II, BD Biosciences). Data were analyzed using FlowJo software. A complete list of antibodies used in this study is provided in the *Supplemental Materials*.

### Proliferation of T cells and B cells

The proliferation of T cells and B cells was examined as described previously.^19^ Briefly, peripheral blood mononuclear cells were incubated with 1 carboxyfluorescein succinimidyl ester (CFSE; 1.25 μL/mL) (Invitrogen/ThermoFisher Scientific) at 37 °C for 10 min. The cells were then washed twice along with 5 mL (cooled to 4 °C) Roswell Park Memorial Institute (RPMI) medium containing 10% fetal bovine serum. Cells were then resuspended in 600 μL of RPMI/10% fetal bovine serum and seeded into 96-well plates with 5 μg/mL phytohemagglutinin, 10 μg/mL lectin from pokeweed mitogen, and the same volume of RPMI. Cells were cultured for 72 h and then stained with CD3-PerCP (clone: HIT3a, BioLegend), CD4-PE-Cy7 (clone: RPA-T4, BioLegend), CD8-PE (clone: RPA-T8, BioLegend), and CD19-APC (clone: HIB19, BioLegend) antibodies, prior to analysis on a FACSCanto II flow cytometer.

### Western blot of p-STAT5

Peripheral blood mononuclear cells were stimulated with IL-2 (200 ng/mL; PeproTech) at 37 °C for 0, 15, 30, or 120 min and then lysed in lysis buffer containing 50 mM HEPES, 10 mM MgCl2, and 140 mM NaCl (pH 8), and supplemented with 0.1% Tween-20, 1% n-dodecylbeta-D-maltoside, 2 mM phenylmethylsulfonyl fluoride, proteinase inhibitors, and phosphatase inhibitors (all from Sigma-Aldrich, St. Louis, MO, USA). The lysates were vortexed for 5 s every 10 min, incubated for 30 min on ice, and centrifuged at 12,000 *g* at 4 °C for 20 min. Supernatants were diluted 1:4 with 5 × loading buffer and heated to 100 °C for 10 min. Proteins were separated by SDS-PAGE and transferred to nitrocellulose membranes (Bio-Rad, Hercules, CA, USA), which were then blocked in 5% low-fat bovine milk in tris-buffered saline/0.05% Tween-20 at room temperature for 1 h. The primary antibody specific for phospho-STAT5 (Tyr694) (clone: D47E7; Cell Signaling Technologies, Danvers, MA, USA) was used at a dilution of 1:1000 dilution and that specific for GAPDH (a horseradish peroxidase (HRP)-conjugated mouse mAb; Proteintech) was used at dilution of 1:2000. The HRP-conjugated goat anti-rabbit IgG secondary antibody (ZSGB BIO, Beijing, China) was used at a dilution of 1:5000. Quantitative analysis of the bands on the western blots was performed using ImageJ (National Institutes of Health, Rockville, MD, USA).

### Vector production and titration

The LV vectors, pCCL-pEF1α-IL2RG.wt-WPRE∗ (EF1a-IL2RG.wt), and pCCL-pEF1α-IL2RG.co-WPRE∗ (EFS-IL2RG.co), were designed and generated at University College London (kindly provided by Prof. Adrian Thrasher). Vectors were packaged by triple transfection of HEK293T cells with the CMV-GAG/POL, CMV-REV, and CMV-VSV-G plasmids cultured in 225 cm^2^ tissue culture flasks. Supernatants were collected at 48 h post-transfection, concentrated by ultra-centrifugation, and resuspended in X-VIVO 15/20 medium (Lonza, Basel, Switzerland). The viral titer was calculated by transducing HEK293 cells with the serially diluted viral preparation, followed by quantitative PCR. The vector for large-scale transplantation and future clinical trials was packaged according to the guidelines in the “Good Manufacturing Practice (GMP) workshop” published by Yiming CMO company (Shandong Province, China) with a GMP virus package license.

### Transduction of ED7R cells

The ED7R cell line deficient in IL2RG was grown in RPMI-1640 medium supplemented with 10% fetal bovine serum, penicillin/streptomycin, and GlutaMax (all from Gibco, Thermo Fisher Scientific, USA). ED7R cells (5 × 10^5^) were transduced for 6 h at a multiplicity of infection (MOI) of 0.625–10 with polybrene (4 mg/mL, Sigma-Aldrich) and maintained in culture for 12 days. Expression of IL2RG protein was analyzed by flow cytometry and that of *IL2RG* mRNA was analyzed by quantitative PCR.

### Transduction of human hematopoietic stem and progenitor cells

CD34^+^ cells were isolated from the BM on a small scale using magnetic beads (CD34 MicroBead Kit 130-046-702; Miltenyi Biotec, Nordrhein-Westfalen, Germany), or from G-CSF-mobilized peripheral blood mononuclear cells provided by the Children's Hospital of Chongqing Medical University (Chongqing, China; Approval No. 2016.132) or the Allcells company (Shanghai, China). CD34^+^ cells were immunoselected using the CliniMACS system (Miltenyi Biotec), pre-activated by culture in X-vivo 20 (Lonza) containing 100 ng/mL human stem cell factor (SCF; PeproTech), 100 ng/mL human Flt-3 ligand (PeproTech), 100 ng/mL human thrombopoietin (TPO; PeproTech), and 20 ng/mL human IL-3 (PeproTech) overnight, and then transduced at different MOIs for 24 h in the same medium in the presence of 4 mg/mL protamine sulfate (Sigma-Aldrich) and 1:100 LentiBOOST (Sirion Biotech, Munich, Germany). After transduction, cells were washed and maintained in liquid culture in activation medium, or grown as individual progenitors in semi-solid Methocult medium (H3434, STEMCELL Technologies, Canada) for two weeks.

### Analysis of vector copy number (VCN) and IL2RG mRNA expression

Genomic DNA was extracted from transduced cells expanded in liquid culture using the QIAamp DNA Mini Kit (Qiagen GmbH, Germany). DNA from colonies was extracted after lysis with proteinase K (Thermo Fisher Scientific) under standard conditions. The mean VCN per cell was analyzed by TaqMan quantitative PCR using primers and probes that annealed to the HIV *psi* sequence and a reference gene (human *ALB* or murine *Ttn*). Results were calculated from a standard curve generated using a plasmid containing the two sequences as a template. All PCR measurements were performed at least in duplicate using a Bio-Rad, T100 Thermal Cycler (Bio-Rad Laboratories, California, USA). Total RNA was extracted using the Total RNA Miniprep Kit (Axygen, China), reverse transcribed into cDNA using the EvoScript Universal cDNA Master kit (Roche Diagnostics GmbH, Germany), and amplified using TaqMan Universal PCR Master Mix (Thermo Fisher Scientific) on a Bio-Rad T100 Thermal Cycler (Bio-Rad Laboratories). Vector-derived *IL2RG* mRNA was measured (in duplicate) by quantitative reverse-transcription PCR using primers that annealed to *WPRE*; human *TFIID* was used for normalization. All primers and probes are listed in the *Supplemental Materials*.

### Oncogenic analysis of transduced CD34^+^ cells in the axilla of nude mice

Human CD34^+^ cells transduced on a large scale with the SIN-EFS-IL2RG.co vectors were injected into the axilla of 5-week-old nude mice (1.5 × 10^6^ per mouse, *n* = 10). HeLa cells (1.0 × 10^6^ per mouse, *n* = 10) were used as a positive control, and an equal volume of phosphate buffer saline solution (0.2 mL per mouse, *n* = 10) was injected as a negative control. The sizes of the nodules and tumors formed at the injection sites and in the organs were measured every two weeks until 65 days post-injection. Mouse body weight was recorded twice per week until sacrifice or 120 days post-injection. Histopathological evaluation of various organs (including the injection site, heart, lung, liver, spleen, kidney, brain, lymphocyte, and any suspicious nodule or lump) was conducted following mouse sacrifice. Oncogenicity analysis was done by the National Center for Safety Evaluation of Drug in accordance with international guidelines for the care and use of laboratory animals (N2021048).

### Statistical analysis

Statistical analyses were performed using GraphPad Prism v.9.0 for Windows. Results were reported as mean ± standard error of the mean. Statistical differences between means were evaluated using the Mann-Whitney or *χ*^2^ test, as appropriate. Differences were considered significant at *P* < 0.05.

## Results

### Diverse clinical and molecular characteristics of newly identified patients with X-SCID

**Figure 1:**
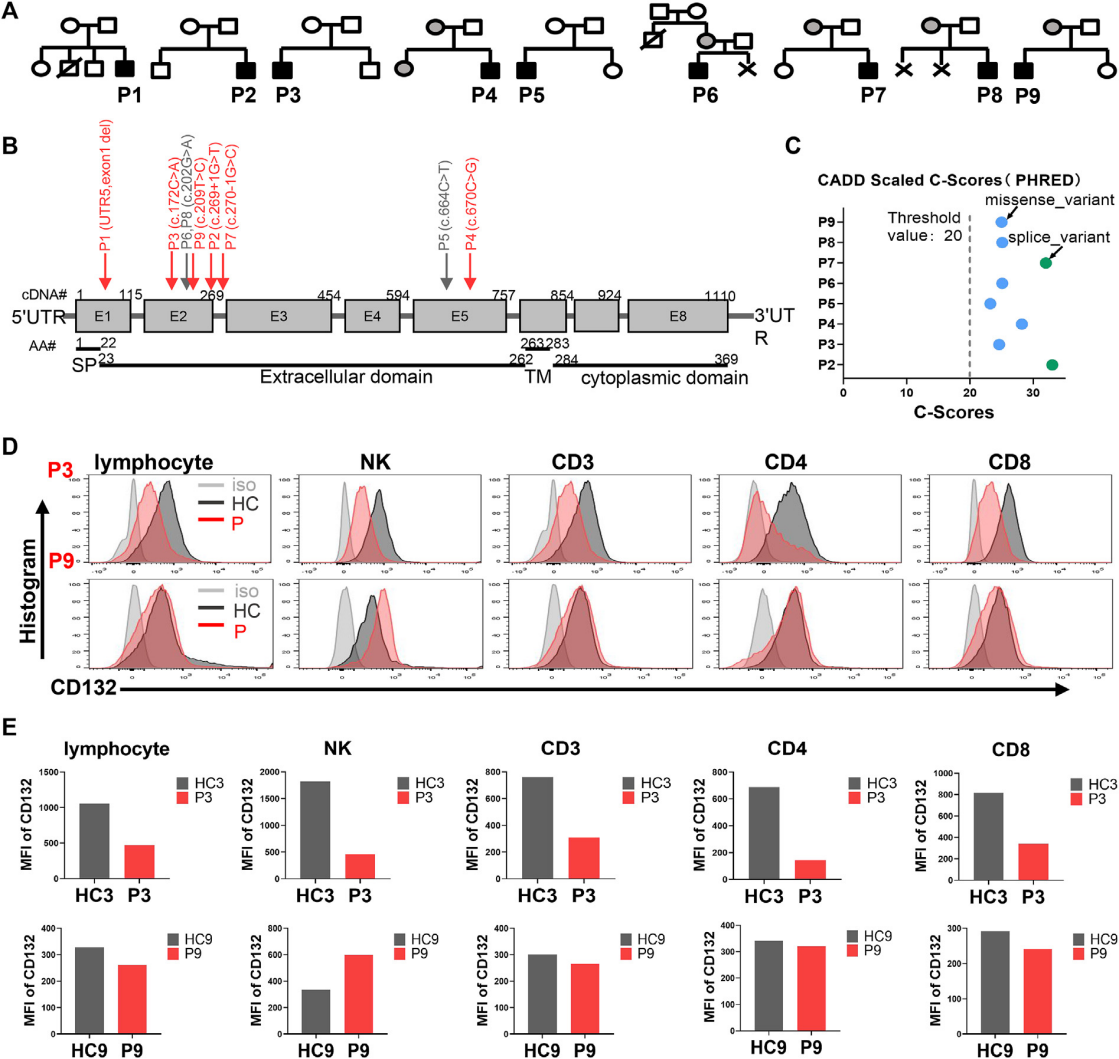
Clinical and molecular characteristics of patients with X-linked severe combined immunodeficiency disease (X-SCID). **(A)** Family pedigrees of the patients. The black solid symbols indicate the affected patients (P1–P9), the grey solid symbols indicate carriers of the same gene mutation, and the open symbols indicate unaffected family members. The squares indicate male subjects and the circles indicate female subjects. The crosses indicate spontaneous abortion or induced abortion because of abnormal prenatal examination and the slashes indicate death at a very young age. **(B)** Schematic representation of *IL2RG* gene map and the protein domain. The grey squares represent the exons (E1–E8). The red arrows indicate novel mutations identified in our patients and the grey arrows indicate previously reported gene mutations in P5, P6, and P8. **(C)** Scaled C-scores of the mutation identified in our patients. The blue dots represent missense variants and the green dots splice variants. **(D)** CD132 expression of total lymphocytes, NK cells, and T cells. The light grey indicates the isotype control, the dark grey shadow indicates the healthy control, and the red shadow indicates the patients. **(E)** Mean fluorescence intensity (MFI) of CD132 in lymphocytes, NK cells, and T cells.

Nine patients attending our hospital were identified as suitable for inclusion (Table 1 and Fig. 1A). All patients were male, and in seven cases, disease onset and diagnosis occurred in their first year of life; all seven infant patients suffered from recurrent severe pneumonia and four had disseminated Bacillus Calmette-Guérin (BCG) disease, while two had meningitis. *Pseudomonas aeruginosa* infection was found in patient 4 (P4). The remaining two patients (P3 and P9) were diagnosed at the ages of 9 and 16 years of age. The exact time of first disease onset in the two remaining patients (P3 and P9) was difficult to ascertain, but both had been suffering from recurrent coughs over 4–5 years prior to their first visit to our hospital (at 9 and 16 years, respectively). They required a ventilator to help them breathe, and there was evidence of bacterial, viral, and fungus infections. Computed tomography scans revealed that both had bronchiectasis. Immunodeficiency was suspected, and immune function screening was performed. Next-generation screening and Sanger sequencing verified mutations in the *IL2RG* gene.

**Table 1:** Clinical and molecular characteristics of newly-identified patients with X-SCID

	P1	P2	P3	P4	P5	P6	P7	P8	P9
Diagnosis age	4m	3m	9y4m	7m	4m	12m	7m	6m	16y
Clinical presentation	Pneumonia, Sepsis, Meningitis	Pneumonia, Disseminated BCG disease, Pneumocystis infection	Pneumonia, Chronic pulmonary fibrosis, Bronchiectasis	Pneumonia, *Pseudomonas aeruginosa* infection	Pneumonia, Skin infection	Pneumonia, Disseminated BCG disease, Inflammatory bowel disease	Pneumonia, Prolonged diarrhea, Disseminated BCG disease	Pneumonia, Disseminated BCG disease	Pneumonia, Bronchiectasis
Gene mutation	UTR5, exon1 del	c.269+1G＞T (IVS2)	c.172C＞A (p.P58T)	c.670C＞G (p.R224W)	c.664C＞T (p.R222C)	c.202G > A (p.E68K)	c.270-1G > C (splicing)	c.202G > A (p.E68K)	c.209T > C (p.M70T)
Novel	Yes	Yes	Yes	Yes	No	No	Yes	No	Yes
De novo	Yes	Yes	Yes	No	Yes	No	No	No	No
Lymphocyte (∗10ˆ9/L)	0.79	0.94	7.31	0.82	4.3	3.44	0.2	2.42	2.28
Immune phenotype	T^−^B ^+^ NK^low^	T^−^B^+^NK^-^	T^+^B^+^NK^+^	T^−^B^low^NK^-^	T^low^B ^+^ NK^+^	T^+^B^+^NK^low^	T^−^B^+^NK^-^	T^+^B^+^NK^-^	T ^+^ B^low^NK^low^
Maternal cell engraftment	NA	NA	No	Yes	No	Yes	Yes	Yes	No
CD132 expression	T (−) B (−) NK (−)	T (−) B (−) NK (−)	T (↓) B (↓) NK (↓)	T (↓) B (−) NK (↓)	T (↓) B (↓) NK (↓)	T (+) B (↓) NK (+)	T (↓) B (↓) NK (↓)	T (↓) B (↓) NK (↓)	T (+) B (↓) NK (+)
Trecs	0	0	4	0	0	0	0	0	1
Krecs	296	476	156	111	588	NA	NA	NA	26
Outcome	Dead	Dead	Alive	HSCT, alive	Alive	HSCT, dead	HSCT, alive	Dead	Alive

BCG: Bacillus Calmette-Guérin, NA: not available, Trecs: T cell Receptor Excision Circles, Krecs: K -deleting recombination excision circles, HSCT: hematopoietic stem cell transplantation, -: absent, ↓: decrease, +: normal.

Eight hemizygous mutations in *IL2RG*, including four *de novo* mutations, were detected in these patients by next-generation sequencing (Fig. 1B). Among the eight mutations detected, six were novel: UTR5 exon1 del (P1), c.269+1G > T (P2), c.17 2C > A (P3), c.670C > G (P4), c.240-1G > C (P7), and c.209T > C (P9). All mutations were suspected disease predicted by CADD (Fig. 1C). The C-score of all eight mutations was all higher than the normal threshold value, particularly for the two splice mutations.

Lymphocyte counts in most patients with X-SCID were <300 × 10^6^/μL, and the immune phenotypes were variable. Two patients (P6 and P8) with the previously reported c.202G > A mutation ^20^ showed similar, but not identical, immunophenotypes; both had a T^+^B^+^ phenotype with maternal engraftment, but P6 had a low number of NK cells. No NK cells were detected in P8. This difference may reflect variations in the level of maternal cell chimerism between these patients. P5 harboring the c.664C > A mutation had a T^low^B^+^NK^+^ immunophenotype, which is consistent with a previous report.^21^ With respect to the remaining patients with novel mutations, P2 with c.269+1G > T and P7 with c.240-1G > C had a typical T^–^B^+^NK^–^ immunophenotype, while P1, who had an exon1 deletion mutation, had an atypical T^–^B^+^ NK^low^ immunophenotype; however, no chimerism data were available for this patient, making it difficult to determine whether the atypical immune phenotype was related to maternal chimerism. P4 with the novel mutation c.670C＞G (p.R224W) also had a typical T^–^B^low^NK^–^ immunophenotype, with 7.4 % maternal chimerism.

**Figure 2:**
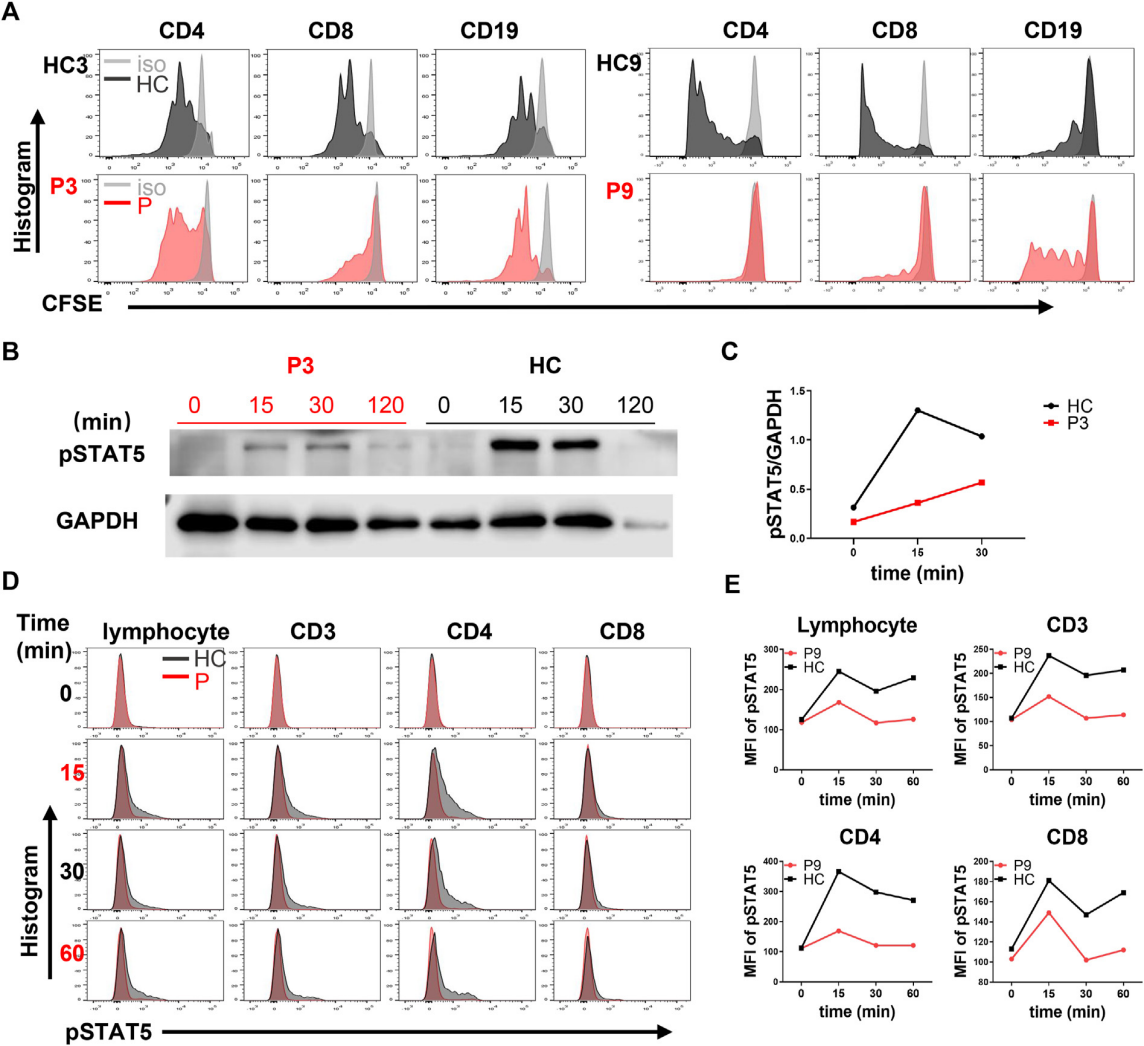
Cell proliferation and STAT5 phosphorylation in P3 and P9. **(A)** A profound T cell proliferation defect in P9 but a slight proliferation defect in P3. **(B)** Western blot of phospho-STAT5 (pSTAT5) expression in peripheral blood mononuclear cells of P3 and HC after stimulation with IL-2. **(C)** The ratio of p-STAT5 to GAPDH was calculated after measuring the band densities of p-STAT5 and GAPDH using ImageJ. **(D)** pSTAT5 expression in lymphocytes and T cells of P9 and HC after stimulation with IL-2. **(E)** MFI of pSTAT5 in lymphocytes and T cells. The light grey indicates the isotype control (iso), the dark grey indicates the healthy control (HC), and the red indicates the patients (P). HC, healthy control; P, patient; MFI, mean fluorescence intensity.

No maternal cells were detected in the two adolescent patients with normal T cell counts (P3 and P9). B and NK cell counts in P3 were normal, but were suppressed slightly in P9. Expression of CD132 fell markedly in P3 but was near-normal in P9 (Fig. 1D, E). In addition, we explored the profound T-cell proliferation defects in P9 and the slight proliferation defect in P3 (Fig. 2A). We found that STAT5 phosphorylation decreased significantly in both CD4 and CD8 cells after activation by IL-2 (Fig. 2B–E). Trecs were absent from almost all patients (Table 1).

At the last follow-up, four of the nine patients had died, including P6, who was treated with HSCT at the age of 1.5 years; this patient died of severe tubercular meningitis at six months post-transplant. The other three died of severe refractory infections and multiple organ failure before they could undergo HSCT. Of those patients who remain alive, only two underwent HSCT. The two older patients accepted intermittent intravenous immunoglobulin replacement therapy, and both suffered from refractory recurrent chronic pneumonia every year.

### Comparison of the EFS-IL2RG.wt and EFS-IL2RG.co vectors in human ED7R cells and BM stem cells

**Figure 3:**
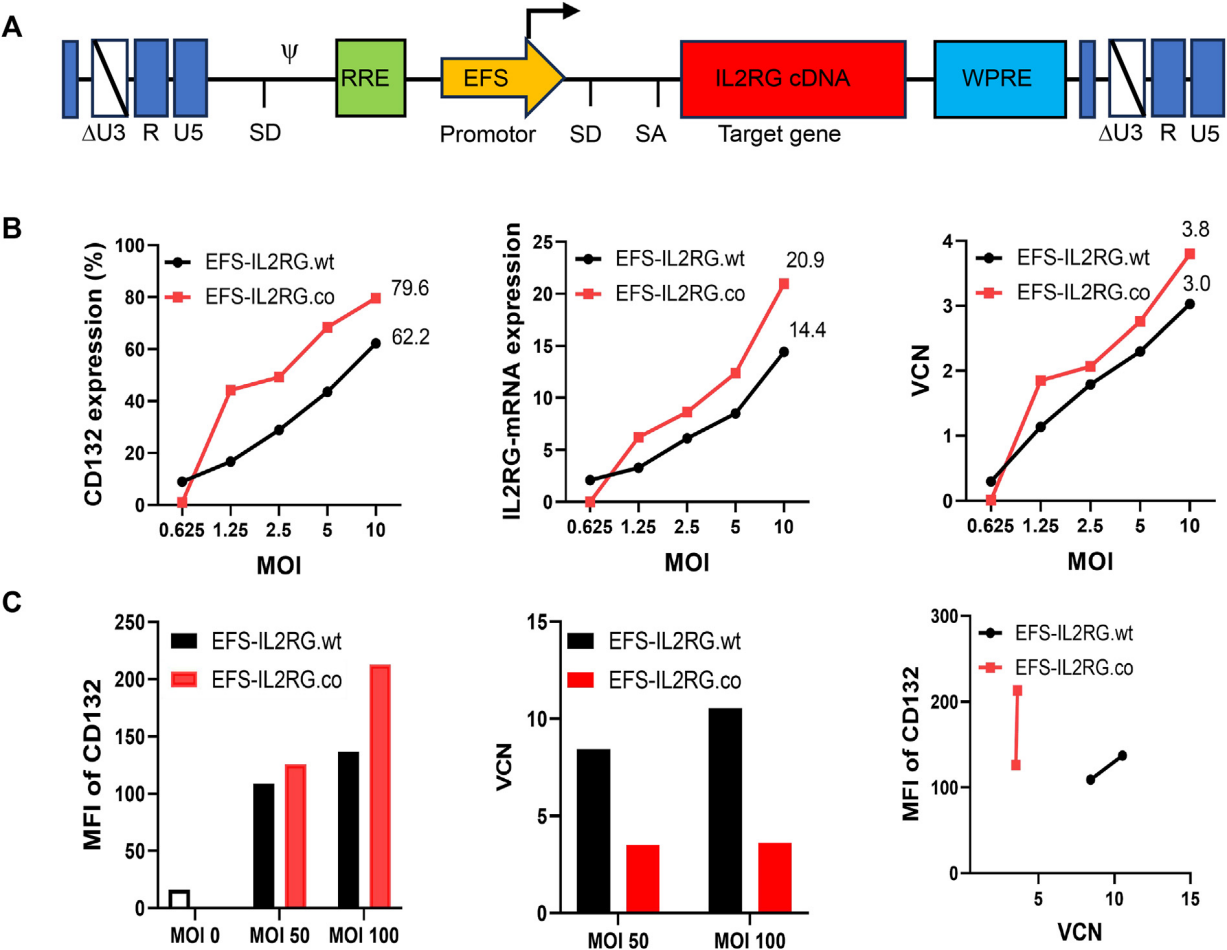
Comparison of the EFS-IL2RG.wt and EFS-IL2RG.co vectors in human ED7R cells and the bone marrow stem cells. **(A)** Scheme of the self-inactivating (SIN) lentiviral vector containing the deleted long terminal repeats (LTRs) (Δ), the short EF1α promoter (EFS), the codon-optimized or wild type version of the IL2RG (IL2RG.co/IL2RG.wt), the HIV-1 Rev Responsive Element (RRE), and a mutated Woodchuck Post-transcriptional Response Element (WPRE). **(B)** Flow cytometry of CD132 expression, IL2RG-mRNA expression, and quantitative PCR analysis of VCN in EFS-IL2RG.co and EFS-IL2RG.wt vector-transduced ED7R cells. IL2RG-mRNA expression is expressed as fold change with respect to endogenous IL2RG in Jurkat cells. **(C)** CD132 expression and VCN in EFS-IL2RG.co and EFS-IL2RG.wt vector-transduced human bone marrow-derived CD34^+^ cells. VCN, vector copy number; MFI, mean fluorescence intensity; MOI, multiplicity of infection.

We constructed SIN lentiviral vectors that were similar to those used in existing global gene therapy trials. Both naive/wild-type (IL2RG.wt) and codon-optimized (IL2RG.co) *IL2RG* cDNA sequences were placed under the transcriptional control of the *EFS* promoter and incorporated a mutated WPRE∗. The sequences of the codon-optimized IL2-RG were designed and synthesized by GeneArt (Fig. 3A). The EFS-IL2RG.wt and EFS-IL2RG.co vectors were produced at a titer of 0.5–1 × 10^10^ IU/mL.

The efficiency of the vectors in driving expression of IL2RG mRNA and protein expression was first tested using T cell leukemia cell line ED7R cells deficient in IL2RG. The results showed that both vectors (at MOIs arranged from 0.625 to 10) effectively reconstituted expression of IL2RG mRNA and CD132 protein in a dose-dependent manner (Fig. 3B). The EFS-IL2RG.co vector appeared to give rise to a slightly higher expression of IL2RG mRNA and CD132 protein than those from EFS-IL2RG.wt. This is likely the result of a higher VCN in the EFS-IL2RG.co vector shown in Fig. 3B.

Next, we compared the efficiency of these two vectors in human BM CD34^+^ cells, which are the targeting cells for this gene therapy. We found a similar trend (higher expression of CD132 conferred by the EFS-IL2RG.co vector than EFS-IL2RG.wt). However, in contrast to ED7R cells, the VCN in CD34^+^ cells transduced with the EFS-IL2RG.co vector was significantly lower (3.52/cell at an MOI of 50) than that in cells transduced with the EFS-IL2RG.wt vector (8.43/cell at a MOI of 50). This trend was also noted in cells transduced at an MOI of 100 (Fig. 3C). Our data clearly suggested that the EFS-IL2RG.co vector gave rise to a more efficient expression of IL2RG than EFS-IL2RG.wt in CD34^+^ cells.

### Small-scale transduction of EFS-IL2RG.co vector into BM stem cells

**Figure 4:**
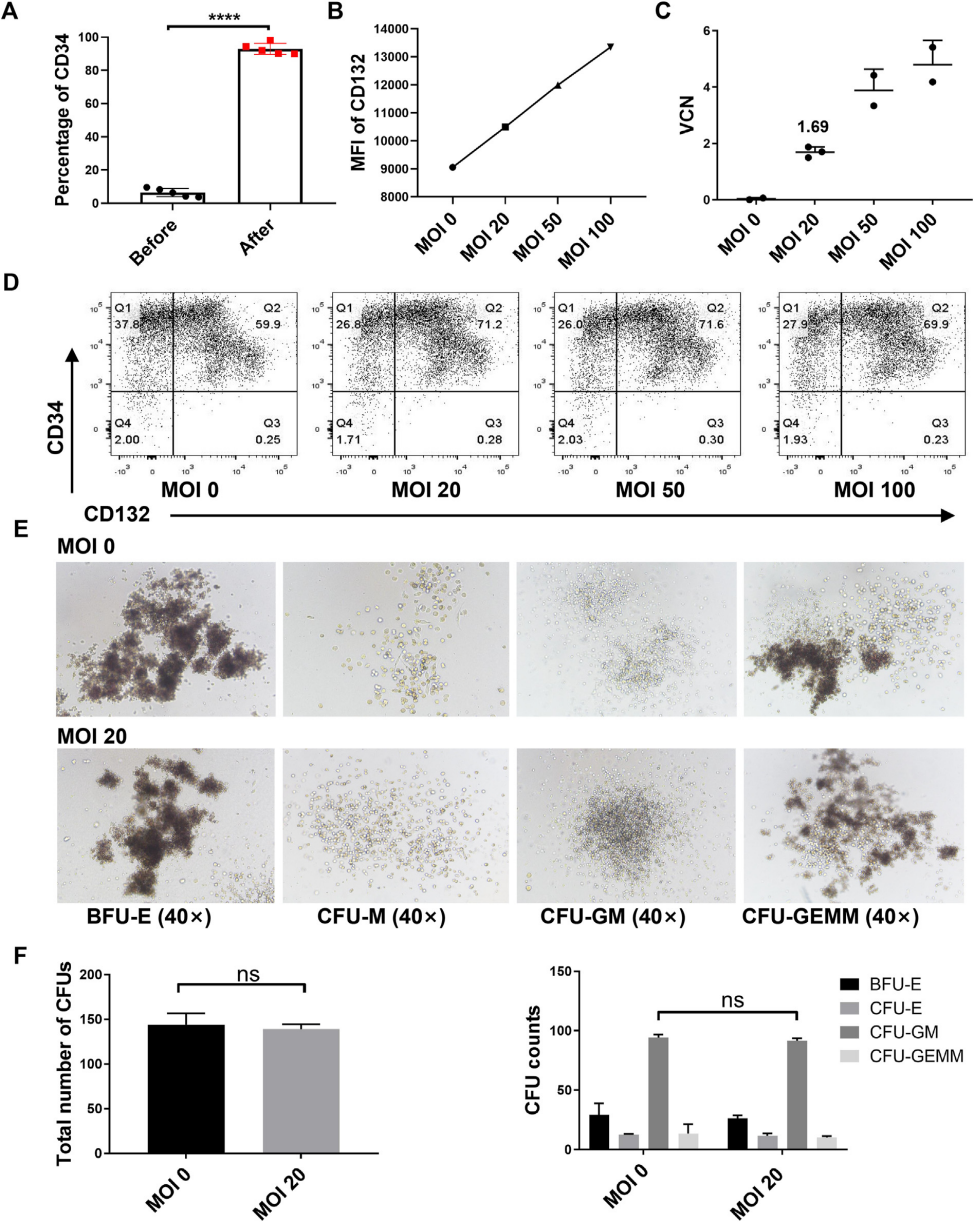
Small-scale transduction of EFS-IL2RG.co vector into bone marrow stem cells. **(A)** The purity of CD34^+^ cells after separation. **(B, D)** CD132 expression in transduced CD34^+^ cells at different MOIs in a small-scale transduction. **(C)** VCN in transduced CD34^+^ cells at different MOIs. **(E)** Hemopoietic cell colonies were observed in all samples from both the transduced and untransduced groups. **(F)** The total numbers of colony-forming units (CFUs) were the same in both groups (left), as were the numbers of colonies of different lineages (right). VCN, vector copy number; MFI, mean fluorescence intensity; MOI, multiplicity of infection.

To further test the EFS-IL2RG.co vector, we isolated CD34^+^ cells from different BM donors using magnetic bead sorting and obtained CD34^+^ cells that were >90% pure after each separation (Fig. 4A). We then transduced CD34^+^ cells at increasing MOIs (20, 50, and 100), and determined the VCN. We found that increased expression of CD132 correlated with the increase in the MOIs (Fig. 4B). The mean VCN (± standard deviation) values in the bulk population of transduced CD34^+^ cells were 1.68 ± 0.19, 3.87 ± 0.76, and 4.80 ± 0.87 at an MOI of 20, 50, and 100, respectively (Fig. 4C). Based on these data, we chose an MOI of 20 for our large-scale transduction study as this is a relevant vector dose required for clinical trials involving X-SCID. Furthermore, the transduced (MOI = 20) and un-transduced CD34^+^ cells were plated in a semi-solid Methocult medium to assess whether they retained the ability to generate multi-lineage hematopoietic progenitor colonies. We found that the total number of colony-forming units (CFUs) was the same in both the transduced (MOI = 20) and untransduced groups, and there was no difference in the numbers of different types/lineages of colonies between the two groups (Fig. 4D, E).

### Large-scale transduction of human mobilized hematopoietic stem and progenitor cells *in vitro*

To mimic the procedures used for the clinical application of gene therapy, large-scale cultures of human mobilized CD34^+^ cells were transduced with the EFS-IL2RG.co vector in the GMP workshop. EFS-IL2RG.co vectors had a titer of 8.9 × 10^9^ TU/mL. A 257 mL apheresis sample was obtained from a healthy volunteer, and 6.57 × 10^8^ CD34^+^ cells were sorted by CliniMACS, with a purity of 98.7%. CD34^+^ cells (1.4 × 10^8^) were stimulated for 20 h, and 1.0 × 10^8^ CD34^+^ cells were transduced for one round (24 h) at an MOI of 20. Samples were taken at each step for quality control testing. Finally, a total of 1.58 × 10^8^ transduced cells were frozen as the final product, and samples were thawed prior to basic quality control tests (Table S3).

**Figure 5:**
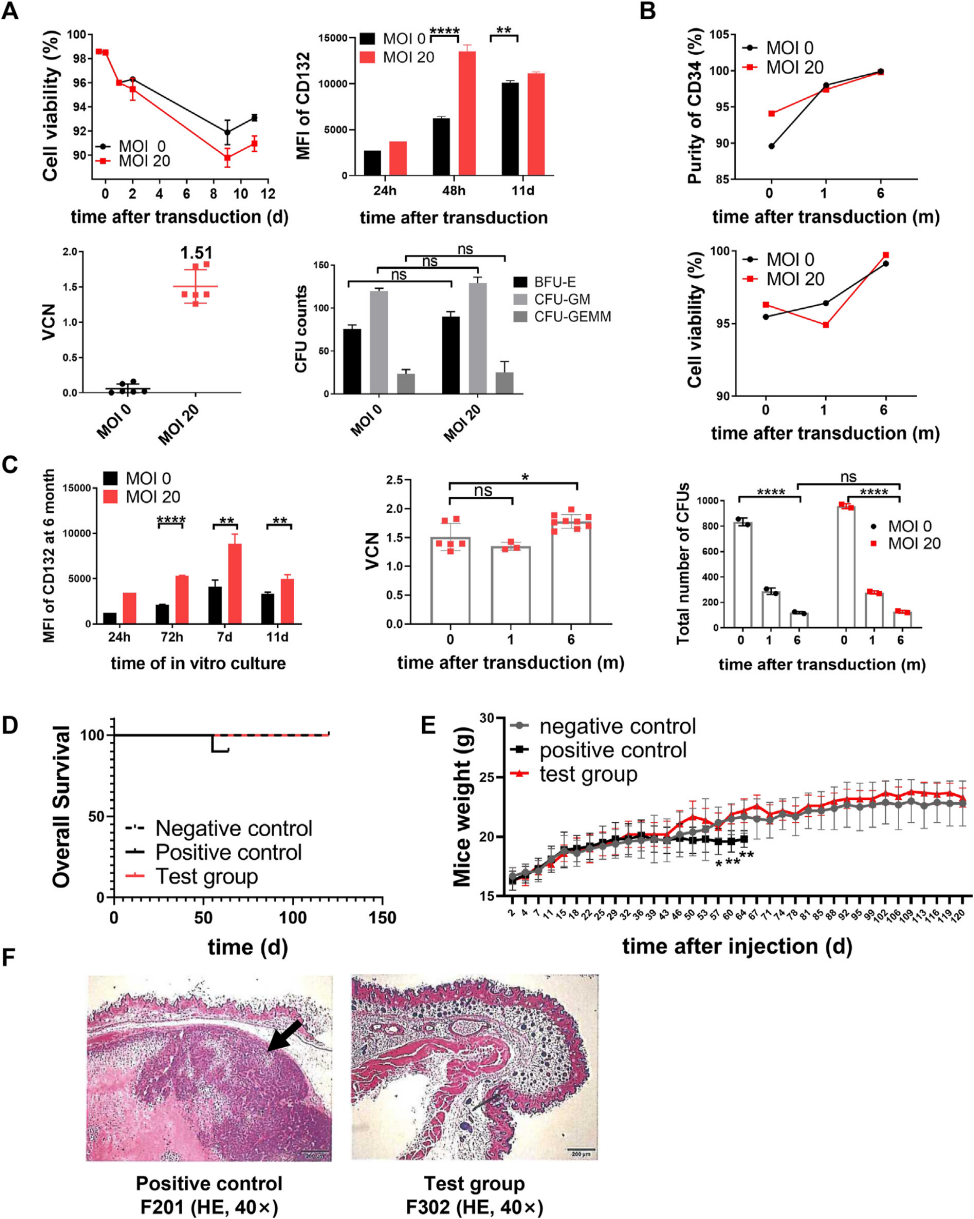
Large-scale transduction of human mobilized hematopoietic stem and progenitor cells *in vitro* and oncogenicity analysis of transduced CD34^+^ cells in nude mice. **(A)** Cell viability of untransduced or EFS-IL2RG.co vector-transduced human CD34^+^ cells in the *ex vivo* culture; the CD132 expression in both untransduced or EFS-IL2RG.co vector-transduced CD34^+^ cells at different time points; VCN in the transduced cells; the numbers of colonies of different lineages formed by untransduced and vector-transduced CD34^+^ cells. **(B)** Cell viability of the final product during re-infusion monitored at different times after thawing. **(C)** The quality check on the frozen large-scale transduced cells at different time points (0, 1, and 6 months). From left to right: The purity of CD34^+^ cells being frozen at 1 and 6 months; the cell viability being frozen at 1 and 6 months; CD132 expression at 1, 3, 7, and 11 days after the transduced cells being frozen at 6 months; VCN and colony forming capacity of the final frozen cell product being frozen at 1 and 6 months. **(D)** Over survival of nude mice in different groups. **(E)** Nude mouse weights in different groups. **(F)** Histopathological evaluation of tumor growth at the cell injection sites in positive control and test group mice. Negative control: nude mice transplanted with an equal volume of phosphate buffer saline solution; positive control: nude mice transplanted with HeLa cells (1.0 × 10^6^ per mouse); test group: nude mice transplanted with human CD34^+^ cells (1.5 × 10^6^ per mouse) transduced with the SIN-EFS-IL2RG.co vector. The data were presented as mean ± standard error of the mean. Statistical differences are expressed as follows: ns, non-significant; ∗∗*p* < 0.01, ∗∗∗*p* < 0.001, and ∗∗∗∗*p* < 0.0001. VCN, vector copy number; MFI, mean fluorescence intensity; MOI, multiplicity of infection.

The results showed that the transduction procedure did not reduce cell viability or the purity of CD34^+^ cells (Fig. 5A–C). The viability of transduced CD34^+^ cells remained at a level similar to that of untransduced cells during 12 days of *ex vivo* culture. Expression of CD132 at day 2 was significantly higher than that in untransduced cells and remained high up until day 12 (Fig. 5A). The VCN at MOI = 20 was at 1.51 (±0.24), which is a similar level to that in the small-scale transduction assay depicted in Figure 4C. In addition, there was no difference observed in the total number of CFUs or types/lineages of colonies.

To further verify the safety and stability of the final product, we assessed and compared the frozen large-scale transduced CD34 cells at different time points. At 0, 30, 60, and 120 min after thawing, cell viability did not change appreciably (Fig. 5B). Additionally, the percentage of viable CD34^+^ cells, CD132 expression levels, and the VCN of the final frozen cell product were comparable at 0, 1, or 6 months post-freezing, demonstrating that freezing for extended periods does not affect the stability of the product; however, CFU counts fell at 1 and 6 months (Fig. 5C). Although the exact reasons for this is not clear (it also occurred in untransduced cells), the data indicate that cell infusion should be scheduled as soon as possible after they are collected *in vitro*.

### Oncogenicity analysis of transduced CD34^+^ cells *in vivo*

To assess the oncogenicity of transduced CD34^+^ cells *in vivo*, we injected the axilla of nude mice with human CD34^+^ cells (1.5 × 10^6^ per mouse) transduced on a large scale with the SIN-EFS-IL2RG.co vector at an MOI = 20. We then recorded tumor growth over time. HeLa cells (1.0 × 10^6^ per mouse) were injected into mice as positive controls and an equal volume of phosphate buffer saline solution (per mouse) was injected into mice as negative controls. The sizes of the nodules and tumors formed at injection sites and in organs were measured every two weeks up until 65 days post-injection. Mouse body weight was recorded twice per week until sacrifice or up until 120 days post-injection. Histopathological evaluation of various organs was conducted following mouse sacrifice.

Overall, the survival rate of mice in the test group was not affected by transplantation with SIN-EFS-IL2RG.co vector-transduced CD34^+^ cells. All mice in the test group were alive at sacrifice (Fig. 5D). The body weight of these mice was comparable with that of mice in the negative group; however, mice injected with HeLa cells lost weight from day 46 post-transplantation, and body weight was much lower than that in the test group after day 57 (*P* < 0.05) (Fig. 5E). There were no signs of tumor growth at the injection sites or in different organs in nude mice injected with transduced human CD34^+^ cells; however, tumors were identified at these sites in the positive group. Histopathology of organs verified that transduced CD34^+^ cells lacked oncogenicity (Fig. 5F).

### The reconstitution of IL2-RG expression on BM stem cells from an X-SCID patient

**Figure 6:**
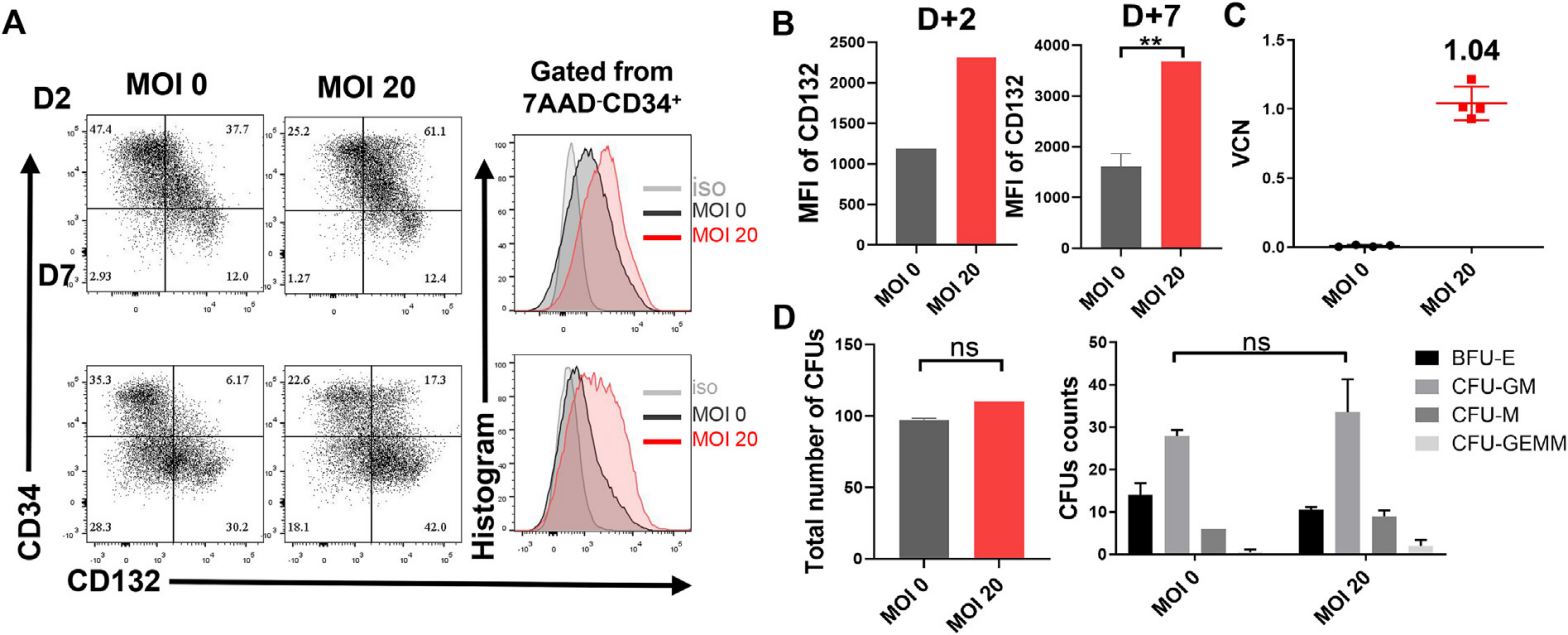
Successful *ex vivo* transduction of CD34^+^ cells from patients with X-linked severe combined immunodeficiency disease (X-SCID). **(A)** CD132 expression in *IL2RG* defect or corrected CD34^+^ cells at 2 days and 7 days after transduction. **(B)** MFI of CD132 in CD34^+^ cells. **(C)** Mean VCNs (± standard deviation) in the bulk population of transduced CD34^+^ cells. **(D)** The total numbers of colony-forming units (CFUs) of CD34^+^ cells in both groups (left) and the numbers of colonies of different lineages (right). The light grey indicates the isotype control (iso), the dark grey indicates the healthy control (HC), and the red indicates patients (P). The data were presented as mean ± standard error of the mean. Statistical differences are expressed as follows: ns, non-significant; ∗∗*p* < 0.01. VCN, vector copy number; MFI, mean fluorescence intensity; MOI, multiplicity of infection.

Finally, we collected a residual clinical BM specimen from P4, and sorted the CD34^+^ cells and transduced them with EFS-IL2RG.co (MOI = 20). CD132 expression and the VCN were measured, and CFUs counted, after two weeks of culture. The total percentage of transduced cells expressing CD132 was >70% and >60% at 2 or 7 days post-transduction, respectively, which is higher than that of untransduced cells (Fig. 6A). The MFI of CD132 was 2-fold higher than that of untransduced cells at 2 or 7 days post-transduction (Fig. 6B). The VCN of transduced cells was 1.04 ± 0.12 (Fig. 6C). Furthermore, the number of CFUs formed by transduced CD34^+^ cells and untransduced cells was the same, indicating that the CD34^+^ cells transduced with EFS-IL2RG.co retain their potential for differentiation (Fig. 6D).

## Discussion

An increasing number of X-SCID cases has been reported, together with information about age of onset, infection profiles, T/B/NK subsets, and gene mutations. The patients in our cohort frequently suffered from BCG disease, likely because national BCG immunization programs begin one month after birth, and there is no newborn screening program in China; this poses a challenge with respect to early diagnosis and management of SCID. Among the novel *IL2RG* gene mutations identified herein, UTR5 exon1 del, c.269+1G > T, and c.240-1G > C are frame-shift or splice-site mutations characterized as directly pathogenic. The missense mutation c.670C > G is also clearly pathogenic since the patient showed a typical T-B + NK cell phenotype; however, the pathogenic effects of c.172C > A(P3) and c.209T > C(P9) pose more of a challenge since they are associated with older age, relatively mild infection, normal T cell counts, and no evidence of maternal cell engraftment. CD132 protein levels were decreased in P3, but normal in P9. Therefore, further functional analysis (*e.g.*, of Trecs and IL-2-activated STAT5 phosphorylation) is essential. Trecs and STAT5 phosphorylation status are evident signs of pathogenesis, which can be used to diagnose X-SCID in atypical cases, even those with normal absolute lymphocyte counts and CD132 expression levels. Identification of these novel mutations expands the genetic variation and clinical warning spectra, which will be very useful in clinical practice.

Three of our patients, including one treated with HSCT, died. Another patient died of meningitis during his first year of life. Among the nine patients, only two were treated successfully with HSCT. The high mortality rate in our X-SCID patients highlights the need to implement newborn screening and gene therapy in China as soon as possible.

Gene therapy for X-SCID was developed during the last century. Moloney murine leukemia virus-based gamma retroviral vectors (γRVs) carrying wild-type IL2RG sequences result in good long-term T cell immune reconstitution and an overall survival rate of 90% (18/20) 7, 8, 9; however, six of the patients developed T cell acute lymphoblastic leukemia 2–14 years after undergoing gene therapy.^10^, ^11^, ^12^, ^13^, ^14^, ^15^ Consequently, a new generation of SIN γRVs and SIN-LVs was developed, and none of the patients treated with these vectors developed leukemia.^22^

The SIN-LV vector used in our study was based on a CCL-SIN-18 LV vector backbone with a codon-optimized *IL2RG* cDNA sequence (IL2RG.co) under the transcriptional control of the *EFS* promoter and mutated WPRE∗.^23^ In our experience, the codon-optimized IL2RG sequences used in our vector produce a higher virus titer (*i.e.*, 2.5 × 10^9^ IU/mL). *In vitro*, the VCN, *IL2RG* mRNA expression, and CD132 levels were higher in ED7R and BM CD34^+^ cells than in the IL2RG.wt vector. This suggests that a lower virus dose is needed to achieve a desirable VCN with the EFS-IL2RG.co vector to reduce the toxicity to CD34 cells, which could potentially be better for clinical application.

We used LentiBOOST to increase transduction efficiency. A published study shows that LentiBOOST increases the transduction efficiency of hematopoietic stem and progenitor cells by 2- to 3-fold.^24^ LentiBOOST and protamine sulfate have been used as “good manufacturing practice-compliant clinical-grade products” for gene therapy. LentiBOOST can increase the total VCN by over 6-fold, with no major changes in global gene expression profiles or inadvertent loss of CD34^+^CD90^+^ hematopoietic stem and progenitor cell populations.^25^ In our study, we verified high-efficiency transduction using LentiBOOST combined with protamine sulfate. The VCN was 1.51 (±0.24) for large-scale transduction of cells at an MOI = 20 when using both LentiBOOST and protamine sulfate, while in the absence of LentiBOOST, an MOI >100 was needed to achieve a VCN of 1.5.

We also collected a residual clinical BM specimen from one patient with X-SCID and tested the transduction efficiency of our EFS-IL2RG.co vector in the context of reconstitution of γ chain on his CD34^+^ cells *in vitro*. The VCN in our transduced cells measured was 1.04 ± 0.12, which is considered suitable for X-SCID gene therapy, and at an MOI of 20, it gave rise to a significantly higher CD132 expression than that in untransduced cells at 2 days and 7 days post-transduction. Furthermore, the transduced CD34^+^ cells retained their stemness and generated a normal number of progeny. We also found that long-term storage of frozen collected cell products may reduce the potential for stem cell differentiation. Thus, cell infusion should be scheduled as soon as possible after cells are collected.

In addition, *ex vivo* gene therapy carried out using the CRISPR/Cas system to correct or replace mutant IL2RG, or to integrate the WT IL2RG into a specific chromosomal site, was explored in preclinical studies and the results are encouraging. As new gene therapy strategies are developed and matured, safety can be further guaranteed.

In conclusion, we demonstrate the efficacy and safety of gene therapy for X-SCID using SIN-EFS-IL2RG.co vector and a method that complies with GMP for preclinical gene therapy requirements. Our study paves the way for clinical trials of X-SCID gene therapy in China. The presence of BCG disease worsened clinical outcomes for our X-SCID patients, demonstrating the necessity of implementing newborn screening and gene therapy as soon as possible in China.

## Ethics declaration

The animals received care in compliance with the Principles of Laboratory Animal Care, and the study was approved by the Ethics Committee of the Children's Hospital of Chongqing Medical University (IACUC Issue No. CHCMU-IACUC20211220001).

## CRediT authorship contribution statement

Yunfei An, Adrian J. Thrasher, Xiaodong Zhao, and Fang Zhang designed this study and revised the manuscript. Mingfeng Hu and Qiling Xu performed the experiments, analyzed the data, followed the patients, and wrote the manuscript. Yelei Gao and Weixia Du helped analyze the data. Karen F. Buckland, Yuan Ding, Lina Zhou, Xiulian Sun, Lijia Ma, Zhiyong Zhang, and Xuemei Tang provided experimental expertise and scientific sights. All authors read and approved the manuscript before publication.

## Funding

This work was supported by the National Natural Science Foundation of China (No. 82070135), the National Key R&D Program of China (No. 2021YFC2700804), and the CQMU Program for Youth Innovation in Future Medicine (China) (No. W0100).

## Conflict of interests

Xiulian Sun was employed by Yunfei An. The remaining authors declare that the research was conducted in the absence of any commercial or financial relationships that could be construed as a potential conflict of interest**.**
