# Supplementary material for: Preclinical ex vivo IL2RG gene therapy using autologous hematopoietic stem cells as an effective and safe treatment for X-linked severe combined immunodeficiency disease
Source: Genes Dis. 2024 Nov 6;12(3):101445. doi: 10.1016/j.gendis.2024.101445 (PMC11907444; doi:10.1016/j.gendis.2024.101445)
Supplement: Multimedia component 1 [file mmc1.docx]

**Table S1 Antibodies used in the study.**

| Antibodies | clone |
| --- | --- |
| h-CD45 | HI30 (invitrogen) |
| h-CD34 | 581 (BD) |
| h-CD132 | TUGh4 (BD) |
| CFSE | C34554 (invitrogen) |
| h-pSTAT5 | D47E7 (CST) |
| m-CD45 | I3/2.3 |
| m-Lineage Cocktail | 145-2C11，M1/70，RA3-6B2，TER-119，RB6-8C5 (BD) |
| m-CD3 | 17A2 |
| m-CD4 | GK1.5 |
| m-CD8 | 53-6.7 |
| m-B220 | RA3-6B2 |
| m-NK1.1 | S17016D |
| m-CD11b | M1/70 |
| m-CD132 | TUGm2 |

**Table S2 primers and probes used in the study.**

| Oligo. name | sequence |
| --- | --- |
| WPRE-Forward | AGGAGTTGTGGCCCGTTGT |
| WPRE- Reverse | TGACAGGTGGTGGCAATGC |
| WPRE- probe | FAM-TGTTTGCTGACGCAACCCCCACT-3’ |
| PSI-Forward | 5’-CAGGACTCGGCTTGCTGAAG |
| PSI-Reverse | 5’-TCCCCCGCTTAATACTGACG |
| PSI-Probe | 5’-FAM-CGCACGGCAAGAGGCGAGG |
| hIL2RG- Forward | CAGGAGACAGGCCACACAGA |
| hIL2RG-Reverse | CACTCAGTTTGTGAAGTGTTAGGTTCT |
| hIL2RG- probe | CY5-CTAAAACTGCAGAATCTGGTGATCCCCTGG |
| hTFIID- Forward | GAGAGCCACGAACCACGG |
| hTFIID- Reverse | ACATCACAGCTCCCCACCAT |
| hTFIID-probe | VIC-TGTGCACAGGAGCCAAGAGTGAAGA-3’ |
| mTtn-Forward | 5’-AAAACGAGCAGTGACGTGAGC-3’ |
| mTtn-Reverse | 5’-TTCAGTCATGCTGCTAGCGC-3’ |
| mTtn-Probe | 5’-VIC-TGCACGGAAGCGTCTCGTCTCAGTC-3’ |

**Table S3 The quality control (QC) tests of the cell product.**

| Items | Test method | Specifications | Results |
| --- | --- | --- | --- |
| Live cell density | Cell count star | (1~10) E+06 cells/ml | 1.90E+06 cells/ml |
| Cell viability | Placental blue staining | 70% | 96.9% |
| CD34 purity | FACS | >90% | 99.8% |
| CD132 expression | FACS | Higher than un-transduced cells | Higher than un-transduced cells |
| PH | PH-meter | 6.00~8.00 | 6.64 |
| Sterility | culture | negative | negative |
| Endotoxin | Sol-gel method | <0.5EU/ml | <0.5EU/ml |
| RCL | Culture | Negative | Negative |
| Mycoplasma | qPCR | Negative | Negative |
| E1A gene transfer | PCR | Negative | Negative |
| SV40 gene transfer | PCR | Negative | Negative |
| Osmotic pressure | Osmometry | 1400~1800 mOsm | 1413 mOsm |
| Appearance | Observation | Colorless suspension | Colorless suspension |
| VCN | qPCR | 1~2 copies/cell | 1.51 copies/cell |
| CFUs | Melt-culture | Reserved differentiation potential | Reserved differentiation potential |

RCL: Replication- Competent Lentivirus, VCN: Vector Copy Number, CFUs: Colony Forming Units.
